# Gerontology Mentoring Across the Career Spectrum: Building an Interdisciplinary Pipeline for the Future

**DOI:** 10.1093/geroni/igaf122.412

**Published:** 2025-12-31

**Authors:** Todd Manini

**Affiliations:** University of Florida, Gainesville, Florida, United States

## Abstract

Mentorship is a critical component of professional development in gerontology, shaping career trajectories from student training to senior leadership. As an inherently interdisciplinary field, gerontology requires mentorship models that span diverse disciplines, professional roles, and career stages. This presentation will explore how structured and informal mentorship can foster interdisciplinary collaboration, support career transitions, and sustain a strong pipeline of gerontology professionals. Drawing from real-world examples and different mentorship philosophies, the presentation will address key strategies for effective mentoring. Special attention will be given to the role of mentorship in attracting practitioners to the field, giving opportunities to early-career professionals to gerontology, and preparing mid-career scholars to serve as mentors themselves. Additionally, the presentation will discuss best practices for mentoring in a remote or hybrid environment, particularly relevant in the post-pandemic landscape of higher education and research. The session will conclude with a discussion on institutional strategies to promote mentorship cultures, including structured programs, and cross-disciplinary collaborations. Attendees will leave with actionable insights on how to enhance mentoring efforts within their own institutions and disciplines, ensuring that the next generation of gerontologists is equipped to address the complex challenges of aging in an interdisciplinary and collaborative manner.

